# Retracing cyanobacteria blooms in the Baltic Sea

**DOI:** 10.1038/s41598-022-14880-w

**Published:** 2022-06-27

**Authors:** U. Löptien, H. Dietze

**Affiliations:** 1 Institute for Archaeoinformatics - Data Science, University of Kiel, Christian-Albrechts-Platz 4, 24118 Kiel, Germany; 2 MIN Faculty, CEN, University of Hamburg, Grindelberg 5, 20144 Hamburg, Germany

**Keywords:** Element cycles, Marine biology

## Abstract

In late summer, massive blooms and surface scums of cyanobacteria emerge regularly in the Baltic Sea. The bacteria can produce toxins and add bioavailable nitrogen fixed from atmospheric nitrogen to an already over-fertilized system. This counteracts management efforts targeted at improving water quality. Despite their critical role, the controls on cyanobacteria blooms are not comprehensively understood yet. This limits the usability of models-based bloom forecasts and projections into our warming future. Here we add to the discussion by combining, for the first time, satellite estimates of cyanobacteria blooms with output of a high-resolution general ocean circulation model and in-situ nutrient observations. We retrace bloom origins and conditions by calculating the trajectories of respective water parcels backwards in time. In an attempt to identify drivers of bloom development, we find that blooms originate and manifest themselves predominantly offshore where conditions are more nutrient-depleted compared to more coastal environments.

## Introduction

Reports of cyanobacteria blooms all over the world have been increasing over the years^1^. This also applies to the Baltic Sea where actual trends in late-summer bloom occurrences (predominantly *Nodularia spumingena*) are, however, discussed controversially^2–7^. Related to such cyanobacteria blooms, toxins might enter into the food web^8,9^ and bioavailable nitrogen (bioavailable N) is added to the system by their ability to fix atmospheric nitrogen^10^. This input by nitrogen fixation is substantial (up to 40% compared to the overall nitrogen loads from rivers and atmospheric deposition^11,12^) and fertilises additional primary production and associated export of organic matter to depth. The latter, in turn, is associated with increased oxygen consumption at depth. The related oxygen depletion has been shown to release phosphorus from anoxic sediments^13,14^. This can alleviate phosphorus limitation of autotrophic growth at the sun-lit surface as mixing and upwelling closes the positive feedback loop which may accelerate deoxygenation.

In summary, there is concern because cyanobacteria add nutrients to an already over-fertilized ecosystem thereby degrading the water quality^15,16^ and there is the apprehension that this process may accelerate as we move further into the Anthropocene^17^. Thus, efforts targeted at a comprehensive understanding are increasing as the societal relevance becomes more apparent^8,18–22^. Even so, several mechanisms behind the formation of a cyanobacteria bloom are yet to be deciphered^23,24^ and reliable quantitative tools for projections are still under development^25^. We argue that the former must be accomplished in order to reliably assess the role of potentially antagonistic anthropogenically-induced eutrophication^14,26,27^ and climate change^28^.

To date, numerous abiotic and biotic factors have been suggested to promote cyanobacteria blooms^29–33^. As concerns their implementations in numerical models the underlying parametrizations are in large parts based on empirical field correlations in a process where modellers adjust poorly constrained model parameters (e.g. the saturation coefficients for light and nutrients) such that model output aligns with (typically noisy) observational data. Among the positive correlations exploited so far (for e.g. *Nodularia spumigena*) are: (1) weather conditions and photosynthetically active radiation, (2) ambient temperatures exceeding 16$^{\circ }$C and (3) elevated phosphate concentrations concurring with relative nitrate depletion^29,30^. Negative correlations include salinities exceeding 10 PSU^32^ and winds in excess of a moderate breeze. In this context we face an apparent paradox where relatively slow-growing diazotrophs compete for bioavailable phosphorus with faster-growing phythoplankton - while they, ultimately stock up the system with nitrogen that is essential to their competitors which can not utilise atmospheric dinitrogen^34–37^. In numerical models the extinction of diazotrophs (unless downstream of denitrification zones^38^) is prevented by diverse approaches which include factoring in diazotrophic capability to utilize dissolved organic phosphorus (DOP) effectively^39^, to utilize relatively low P-concentrations^40^, and selective grazing where cyanobacteria experiences less grazing pressure than other phytoplankton^41–43^.

The present study adds to the ongoing discussion by merging satellite data, high-resolution ocean circulation modelling and in-situ observations to dissect the bloom-triggering factors. The aim is to overcome problems associated with the (limited) availability of observational data and to include not only the bloom peak but also the bloom formation phase into our analyses. Blooms are highly variable in space and time which translates into a relative sparseness of observational data despite substantial observational effort. Consequently, model evaluations of simulated diazotrophy in the Baltic Sea have mostly been limited to long-term averages or interannual variations of cumulative proxies indicative of standing stocks^43,44^. Our focus is on environmental conditions preceding bloom manifestations (as picked up by satellite) rather than on conditions during blooms (which were already investigated in the past^45,46^). Essentially, this study compares trajectories of water parcels that develop blooms with those that don’t in the Baltic Proper.

## Results

### Tracing the bloom origins

**Figure 1:**
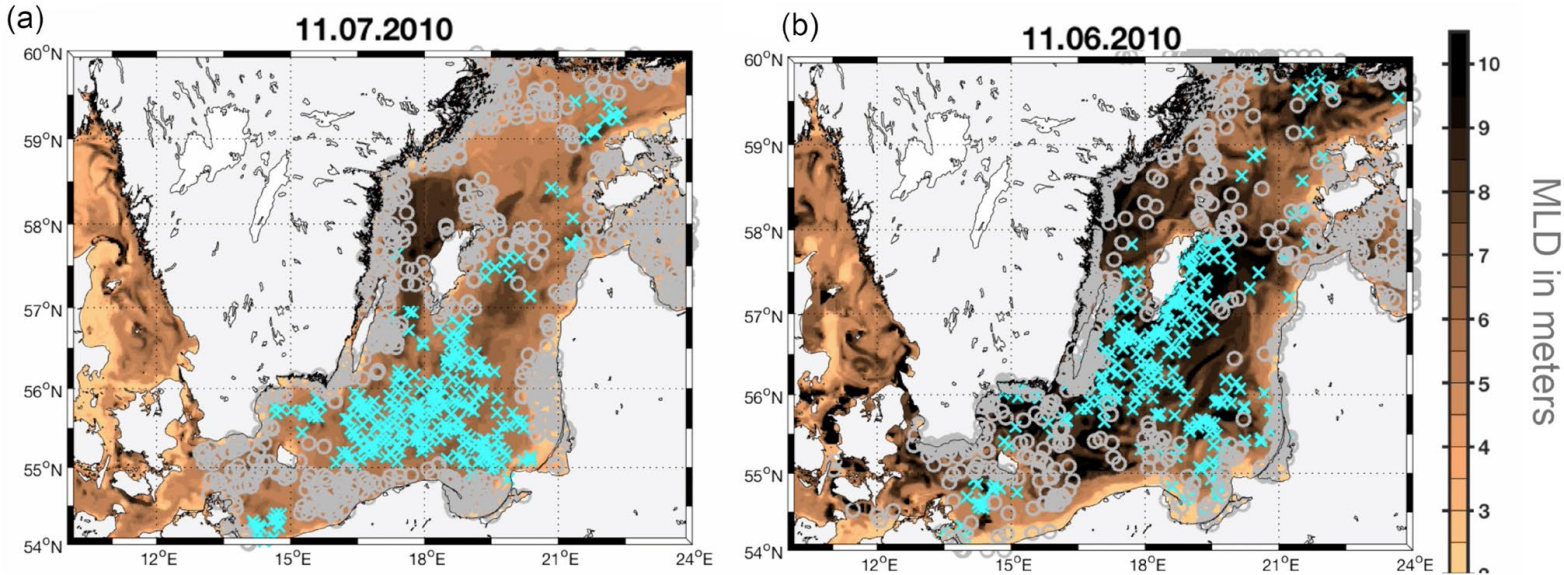
Exemplary back-tracing of a cyanobacteria bloom event. Panel (**a**) shows the random seeding of virtual drifters during a blooming picked up by satellite on July 11th 2010. The crosses (circles) denote drifters seeded into (non-) blooming patches. Unclassified patches are not seeded. Panel (**b**) shows the back-traced positions of the drifters 30 days prior to the blooming event. The color shading indicates surface mixed layer depth in meters. This figure was created with Matlab (https://www.mathworks.com/products/matlab.html).

Figure 1a shows the initialisation for the bloom event identified from space on July 11th, 2010^52^. Randomly seeded drifters (i.e., randomly distributed virtual floats) were attributed to blooming and non-blooming water parcels based on satellite information. Drifters that were seeded at locations of inconclusive satellite information were discarded (further details on this procedure are provided in the Method section). Questions we set out to answer are: What are the differences among bloom-forming water parcels and those that develop no bloom? Do they share a common history of environmental conditions or can we dissect differences hinting at controls of diazotrophy?

### History of water parcels

**Figure 2:**
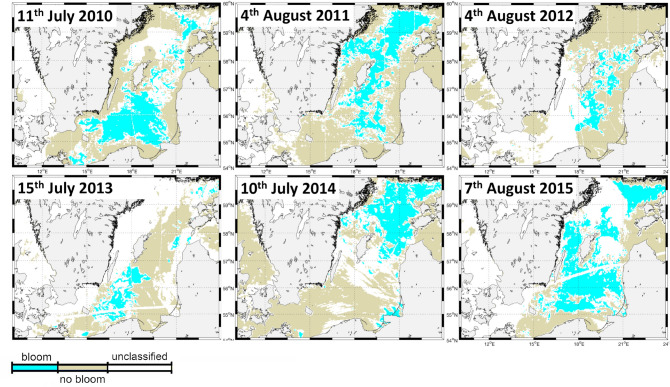
Major cyanobacteria blooms during 2010-2016 for which origins were retraced 60 days backwards in time. Cyan marks grid points where a boom was detected while the beige color marks grid points with no bloom occurrence. White refers to pixels which could not be attributed (due to clouds or uncertain/sub-surface blooms). This figure was created with Matlab (https://www.mathworks.com/products/matlab.html).

The history of water parcels is retraced using the high-resolution general ocean circulation model MOMBA^47,48^. Starting from initial (random) positions trajectories are calculated for 60 days backwards in time using archived MOMBA output of surface currents. We implicitly assume that all blooming is preconditioned within 2 months. Figure 1b exemplarily shows the retraced origin of each water parcel 30 days prior to the blooming event on July 11th, 2010. Respective animations for all events considered here (one per year throughout 2010 to 2015 as shown in Fig. 2) are archived at http://www.baltic-ocean.org/category/cyano.

**Figure 3:**
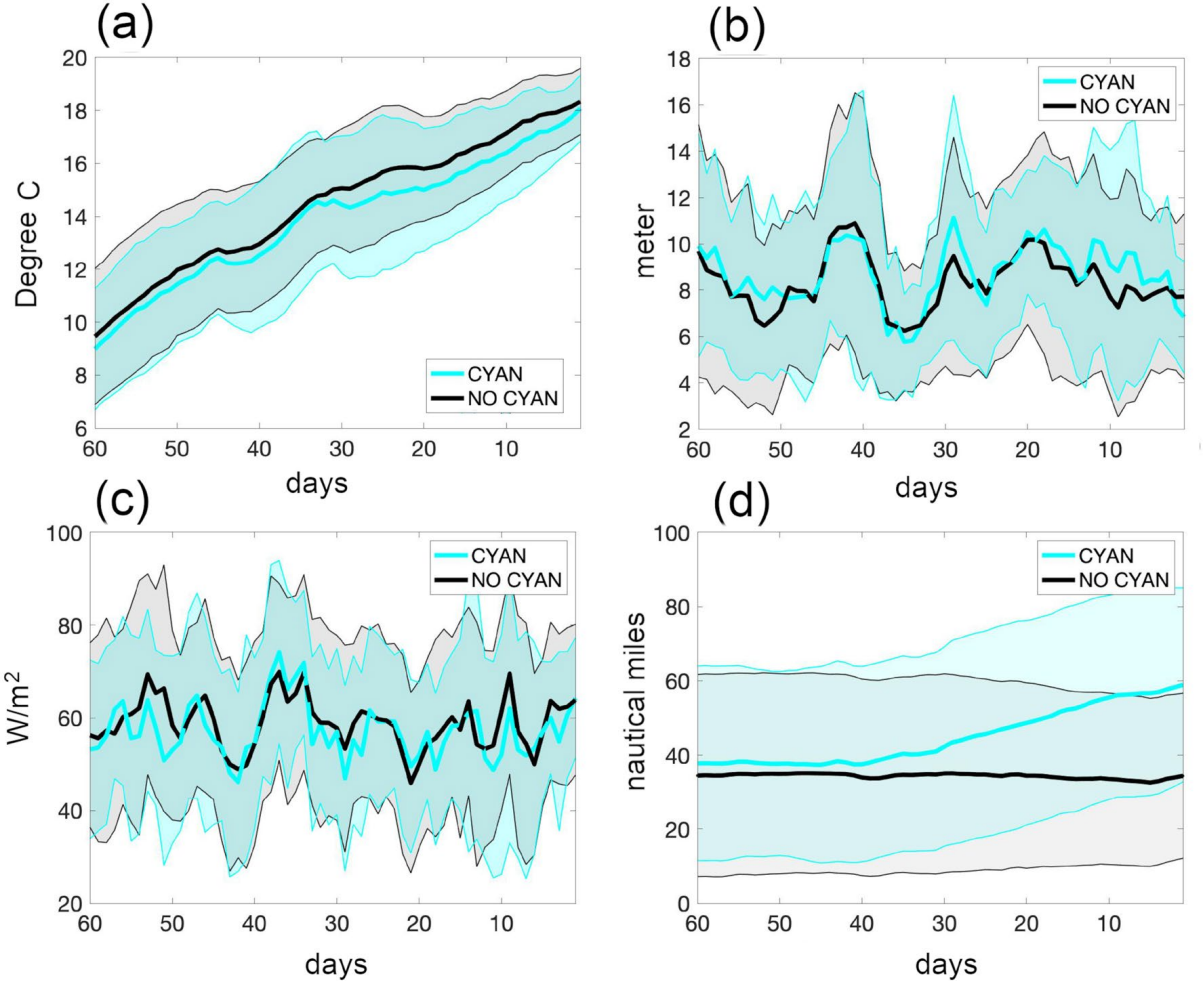
Environmental abiotic conditions relative to bloom events. Cyan colors depict trajectories which later developed blooms. Black and grey trajectories did not develop blooms. Panel (**a**) refers to sea surface temperature, (**b**) to mixed layer depth (MLD), (**c**) to the photosynthetically active radiation (PAR) and (**d**) to the distance to the coast. Curves show are the averages of all blooming and non-blooming trajectories throughout 2010 to 2015 in thick cyan and black lines, respectively. The shaded areas show the respective standard deviations. This figure was created with Matlab (https://www.mathworks.com/products/matlab.html).

Figure 3 summarises the abiotic conditions to which water parcels are exposed throughout the 60 days leading up to a blooming event: Contrary to our expectations we can not identify robust links between sea surface temperatures, photosynthetically active radiation and surface mixed layer depth, on the one hand, and blooming on the other hand. The respective differences among parcel trajectories leading to blooms, and trajectories which do not, are very small and appear insignificant. For example, sea surface temperatures associated with blooms are on average merely $0.25^{\circ }$ colder and do not occur consistently in all of the considered years. Further, photosynthetically active radiation and surface mixed layer do also fail to exhibit consistent differences throughout the 60 day periods.

The surprise, regarding these very small temperatures and mixed layer depths differences, is that cyanobacteria have been shown to grow more optimal in warmer temperatures^25^ and, further, that the high energetic requirements of utilising the exceptionally chemically stable atmospheric dinitrogen molecules suggest a preference for higher radiation levels as given by, e.g., shallower surface mixed layers pervaded by solar radiation (and higher radiation levels). While abiotic environmental conditions such as radiation and temperature that are known to affect physiological processes in cyanobacteria cells^25^ lack all explanatory power to distinguish between blooming and non-blooming water parcels, the distance to the coast is apparently strongly linked to blooming probability: Fig. 3d shows that water parcels that result in blooming separate from the coast and travel offshore. The separation starts weeks prior to blooming which allows enough time for differing environmental conditions to manifest themselves in blooming dynamics. More specifically we find that 90% of blooming water parcels stay more than 17 nautical miles offshore in the 3 weeks prior a bloom. This compares well to the spatial scales of coastal upwelling events which are limited to 15 nautical miles in the Baltic Sea^49^. We interpret this coincidence as evidence for upwelled coastal water preventing blooming. So what is different between near coastal water and conditions offshore?

### Typical nutrient characteristics

**Figure 4:**
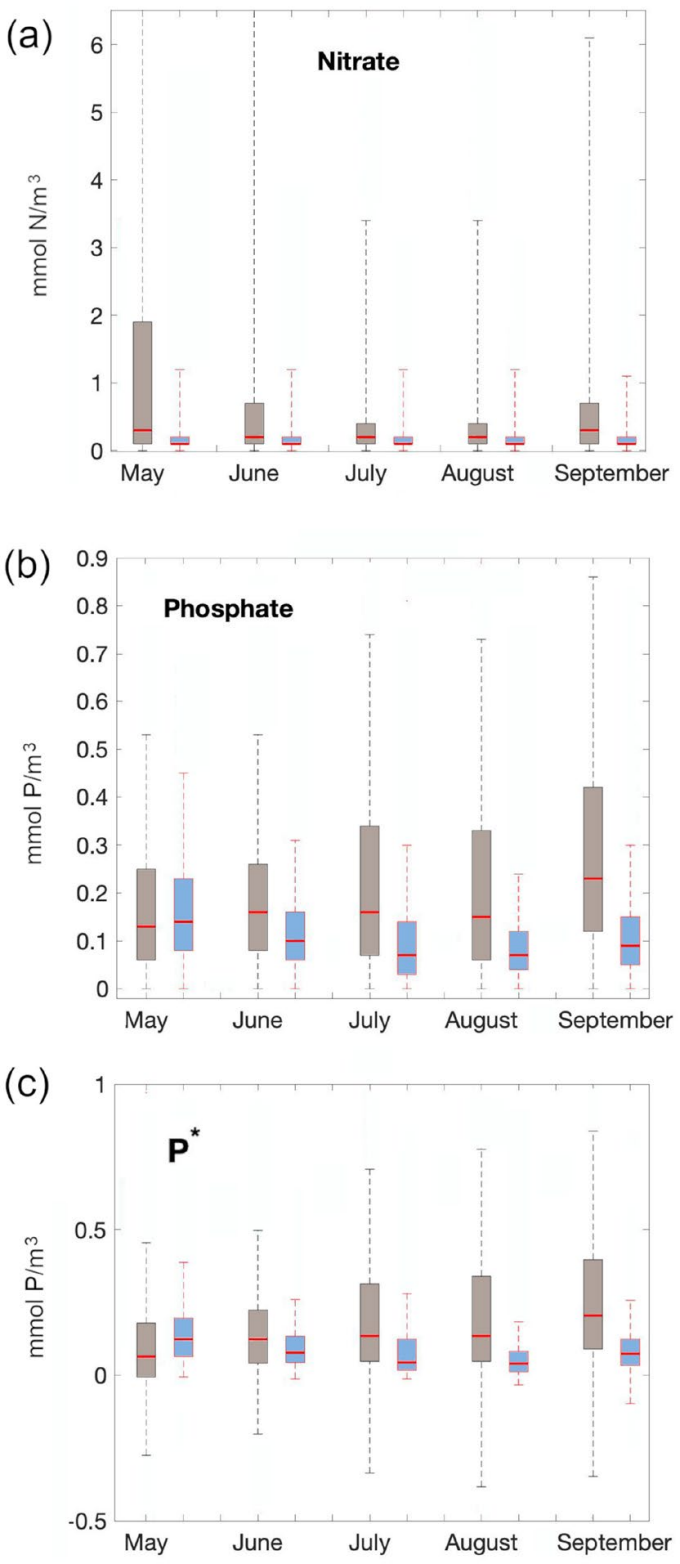
Nutrient concentrations of coastal (brown whisker plots) and off-shore surface waters (blue whisker plots). Panel (**a**) refers to nitrogen, (**b**) to phosphate and (**c**) to P∗ (i.e., phosphate in excess to the P:N Redfield-ratio 1:16). The central red marks indicate the respective medians and the colored squares the 25th and 75th percentiles. The whiskers extend to the most extreme data points (outliers, here defined as exceeding 1.5 times the interquartile range, are discarded). This figure was created with Matlab (https://www.mathworks.com/products/matlab.html).

The biochemical properties of near coastal waters are known to differ from the waters further off-shore^50^. Typically they are more enriched in nutrients that are essential for algae growth because coastal upwelling taps into deep nutrient replete waters and thereby fertilises the sun-lit surface ocean. Figure 4 confirms that offshore waters that are more prone to develop cyanobacteria blooms are characterised by more oligotrophic, nutrient-depleted conditions than the more coastal waters that bloom less: all available (irregular sampled) ICES surface nutrient observations from 1990-2017 are binned into climatological months. Based on our results from Fig. 3 we define coastal distances of more than 60 nautical miles as “off-shore” and distances closer than 40 nautical miles as near “coastal” and find, amidst great variability, that offshore waters are generally more nutrient-depleted than waters closer to the coast. This applies both to nitrate and phosphate surface concentrations except for very early in May.

## Discussion

We set out to find links between environmental conditions and the occurrences of cyanobacteria blooms. Foregoing studies focussed on conditions during the peak of the blooms^45^, or on the frequency of bloom occurrences over the years^46^. In contrast, our approach of combining high-resolution ocean circulation modelling, satellite data, in-situ observations and a back tracing algorithm made it possible to compare the history and trajectories of water parcels comprising a later bloom with the history of “non-blooming” parcels. To this end we add to the discussion on controls of diazotrophy by providing an analysis that is inherently free of seasonal biases and limelights conditions during the growth phase of cyanobacteria in comparison to non-bloom forming waters.

Our results differ from earlier findings that stress the importance of photosynthetically available (solar) radiation and surface mixed layer depth (or wind) for bloom formation. We could also not confirm the hypothesis that blooms may be triggered by strong wind-induced mixing events^51^ - even though our approach was designed to confirm and quantify just these links. Moreover, we found no indication of an influence either of insolation or of temperature other than that blooming occurs generally in summer.

The only apparent difference between bloom forming waters and other water masses in our analysis is the distance to the coast: trajectories of water parcels that develop a bloom are typically situated further offshore than those that do not. Because coastal and offshore waters have different biogeochemical characteristics^50^ this may provide a clue as for what may trigger the blooms. Specifically, we find that average nutrient concentrations of phosphate and nitrate are lower offshore. This applies also to P$^*$ (defined as DIP in excess to DIN/16).

Our results apparently contradict a common assumption that P$^*$ is a major driver of cyanobacteria blooms in the Baltic and we speculate that ecological complexity obscures simple links between physiological traits and manifestations of blooms in field data.

Caveats apply. Among them are uncertainties associated with the satellite retrieval algorithm and the realism of the ocean-circulation model used to back-trace the conditions that led to blooming events.

## Methods

### Observational Data

#### Satellite Data

The bloom detection is based on satellite observations using an automatic routine for bloom classification which is in production at the Swedish Meteorological and Hydrological Institute since 2010. The routine is based on Kahru, MEPS 343:2007 and produces 2-dimensional maps every few days^52^. The underlying data were mainly obtained from the satellites ENVISAT and EOS-AQUA (the MERIS and MODIS sensors). Concerns that detected blooms might mainly refer to surface scums would have been detected when analysing mixed layer depths. Typically only very few larger blooms occur every year. Those large-scale and fully developed blooms form the basis of our analysis. Based on visual inspections to detect these major blooms we chose the following dates: 11th of July 2010, 4th of August 2011, 4th of August 2012, 15th of July 2013, 10th of July 2014, 7th of August 2015 (Fig. 2). The onset of these blooms is generally relatively sudden with only smaller stray bloom patches in the area some days in advance (cf. respective visualizations archived at http://www.baltic-ocean.org/category/cyano).

After identifying the blooms in satellite observations backward trajectories up to 2 months prior to bloom occurrence were calculated. Initial positions of 4000 drifters were randomly seeded within a region bounded by 13$^{\circ }$E to 24$^{\circ }$E and 54$^{\circ }$N to 60$^{\circ }$N. These drifters were then attributed to blooming and non-blooming patches. Patches where the satellite data were inconclusive were discarded. Conditions prevailing within these parcels during their 2 month voyage through the Baltic were analysed and compared for bloom forming and non-bloom forming waters. The respective conditions and analyses are based on a combination of model output for the abiotic factors (i.e. sea surface water temperature, mixed layer depth and photosynthetically active radiation (PAR) within the mixed layer) and observed nutrients.

#### Nutrient observations

 In-situ observations of surface nutrients were provided by the International Council for the Exploration of the Sea (ICES) with a precision of 10$^{-2}$ for phosphate and 10$^{-1}$ for nitrate. Despite the extraordinarily dense data coverage (when compared to typical open ocean conditions e.g. in the Atlantic), the data were still too sparse for robust conclusions based on measurements that were taken in the water parcels that were actually tracked. We mitigated this problem by binning data into: (1) observations during summer seasons 1990-2017 and (2) into near coastal and offshore data. Hereby coastal was defined as waters less than 40 nautical miles off the coast while offshore values refer to all measurements more than 60 nautical miles off the coast. This choice is motivated by Fig. 3d. Overall our approach results in 8146 coastal and 3819 offshore observations for nitrate (for 11148 coastal and 5953 offshore observations for phosphate) during the considered months Mai-September. This choice ensured that our offshore sites were not directly influenced by recently upwelled coastal water because those events have been shown to be limited to 15 nautical miles from the coast into the Baltic Sea^49^.

### The Baltic Sea Model (MOMBA)

 We use a hindcast simulation from 2010 to 2015 integrated with the ocean general circulation model MOMBA for the Baltic Sea to calculate backward trajectories of water parcels hosting (and not hosting) cyanobacteria blooms. The underlying model code and framework is the Modular Ocean Model (MOM), version MOM4p1^53^, coupled to the Sea Ice Simulator (SIS). The Baltic Sea model configuration MOMBA which we use here is documented and assessed in^47^. The model domain covers the entire Baltic Sea and parts of the North Sea (4.2 and 30.3$^{\circ }$E and 53.8 to 66$^{\circ }$N). The model features a horizontal resolution of $\approx$1.9 km (corresponding to one nautical mile) and is eddy-rich in that it starts to resolve the relevant spatial scales^54^. The vertical discretization comprises 47 levels. There are no open boundaries and the model domain is surrounded by solid walls. We use the K-profile parameterization (KPP^55^) with parameters identical to those applied in other eddy-permitting configurations^56,57^. The difference in MOMBA settings relative to^47^ is the atmospheric boundary condition which changed to reanalysis data provided by the Swedish Meteorological and Hydrological Institute based on the atmospheric model RCA4^58^.

### Backward trajectories

 In order to identify the conditions that lead to cyanobacteria blooms (in relation to conditions that do not) we assume that the flow field consists of (Lagrangian) water parcels, each associated with a volume of surface water. 4000 water parcels for analysis are randomly chosen (or seeded) within the central Baltic Sea (ranging from13$^{\circ }$E to 24$^{\circ }$E and 54$^{\circ }$N to 60$^{\circ }$N) during the bloom peaks in 5 consecutive years, identified as described in Sect. 2.1.1. The parcels (or virtual drifters) are divided into two groups - parcels that are located within identified cyanobacteria bloom patches and those who are outside blooming patches. (A third group related to patches where the satellite classification algorithm is inconclusive is discarded). An example of parcel allocation for a bloom in 2010 is depicted in Fig. 2. The trajectory of each water parcel is calculated backward in time from the simulated Eulerian surface velocity fields as calculated by the ocean circulation model MOMBA based on 3-hourly simulated surface velocity model output. For all calculations, the velocities, that are defined on the MOMBA spatial model grid are linearly interpolated in space and time onto the (moving, back-traced) positions of water parcels. Back-tracing is calculated up to 60 days prior to respective blooming using a generic Euler forward (or rather backward) algorithm. Note, that a similar approach to calculate backward trajectories has been used and tested earlier for other purposes and other regions^59,60^.

In a second step, the back-traced trajectories are linked to their respective simulated abiotic water mass properties (such as surface water temperature, mixed layer depth and photosynthetically active radiation (RAR) within the mixed layer). PAR is calculated from the incoming solar radiation averaged over the mixed layer while assuming that PAR provides 42% to the incoming solar radiation and assuming a constant light attenuation of 0.2 $m^{-1}$^61^. As a caveat we report here that shading effects of phytoplankton blooms are not included in this calculation. This effect has been suggested to be important e.g. in lakes whereby cyanobacteria evade shading-effects of ordinary phytoplankton by increasing their buoyancy^62^. In the Baltic Sea, cyanobacteria surface-scums are presumably the major plankton-induced shading events. The latter renders an inclusion of shading effects problematic because causal relationships may be obscured if the presence of cyanobacteria is linked to environmental conditions caused by themselves.

## Data Availability

Movies containing the satellite data and the backtraced drifters are archived in form of movies at http://www.baltic-ocean.org/category/cyano. The tracking data are available at 10.5281/zenodo.6641549. The data are distributed under the Creative Commons Attribution 4.0 License.

## Code availability

The circulation model code MOM4p1 is distributed by NOAA’s Geophysical Fluid Dynamics Laboratory (https://github.com/mom-ocean/MOM4p1, last access: 5 January 2022, Griffies, 2009). MOMBA-specific settings are provided at (https://data.geomar.de/downloads/20.500.12085/900c10df-7b34-4565-b2be-1fff9e801f02/, last access: 19 June 2022).

## References

[1] Kutser T (2009). Passive optical remote sensing of cyanobacteria and other intense phytoplankton blooms in coastal and inland waters. Int. J. Remote Sens..

[2] Kahru, M. *Monitoring algal blooms: New techniques for detecting large-scale environmental changes* Ch.3 (Springer, 1997)

[3] Finni T, Kononen K, Olsonen R, Wallström K (2001). The history of cyanobacterial blooms in the Baltic Sea. AMBIO: J. Human Environ..

[4] Rönnberg C, Bonsdorff E (2004). Baltic Sea eutrophication: Area-specific ecological consequences. Hydrobiologia.

[5] Suikkanen S (2013). Climate change and eutrophication induced shifts in northern summer plankton communities. PLoS ONE.

[6] Kahru M, Elmgren R (2014). Multidecadal time series of satellite-detected accumulations of cyanobacteria in the Baltic Sea. Biogeosciences.

[7] Olofsson M, Suikkanen S, Kobos J, Wasmund N, Karlson B (2020). Basin-specific changes in filamentous cyanobacteria community composition across four decades in the Baltic Sea. Harmful Algae.

[8] Sipiä VO, Kankaanpää HT, Flinkman J, Lahti K, Meriluoto JAO (2001). Time-dependent accumulation of cyanobacteria hepatotoxins in flounders (Platichthys flesus) and mussels (Mytilus edulis) from the northern Baltic Sea. Environ. Toxicol..

[9] Karlsson KM, Kankaanpää H, Huttunen M, Meriluoto J (2005). First observation of microcystin-LR in pelagic cyanobacterial blooms in the northern Baltic Sea. Harmful Algae.

[10] Olofsson M, Klawonn I, Karlson B (2021). Nitrogen fixation estimates for the Baltic Sea indicate high rates for the previously overlooked Bothnian Sea. Ambio.

[11] Larsson U, Hajdu S, Walve J, Elmgren R (2001). Baltic Sea nitrogen fixation estimated from the summer increase in upper mixed layer total nitrogen. Limnol. Oceanogr..

[12] Wasmund N, Voss M, Lochte K (2001). Evidence of nitrogen fixation by non-heterocystous cyanobacteria in the Baltic Sea and re-calculation of a budget of nitrogen fixation. Marine Ecol. Progr. Ser..

[13] Conley DJ, Humborg C, Rahm L, Savchuk OP, Wulff F (2002). Hypoxia in the Baltic Sea and basin-scale changes in phosphorous biogeochemistry. Environ. Sci. Technol..

[14] Vahtera E, Laamanen M, Rintala JM (2007). Use of different phosphorus sources by the bloom-forming cyanobacteria aphanizomenon flos-aquae and nodularia spumigena. Aquatic Microbial Ecol..

[15] Elmgren R, Larson U (2001). Eutrophication in the Baltic Sea area: Integrated coastal management issues.

[16] Nehring D (1992). Eutrophication in the Baltic Sea. Marine Coastal Eutrophication, Proceedings of an International Conference, Bologna Italy.

[17] Vahtera E, Conley DJ, Gustafsson BG, Kuosa H, Pitkanen H, Savchuk OP, Wulff F (2007). Internal ecosystem feedbacks enhance nitrogen-fixing cyanobacteria blooms and complicate management in the Baltic Sea. AMBIO: J. Human Environ..

[18] Stal LJ, Albertano P (2003). Baltic Sea cyanobacteria an investigation of the structure and dynamics of water blooms of cyanobacteria in the Baltic Sea - responses to a changing environment. Cont. Shelf Res..

[19] Hense I (2007). Regulative feedback mechanisms in cyanobacteria-driven systems: A model study. Marine Ecol. Progr. Ser..

[20] Kuznetsov I, Neumann T, Burchard H (2008). Model study on the ecosystem impact of a variable C:N: P ratio for cyanobacteria in the Baltic proper. Ecol. Model..

[21] Hense I, Burchard H (2010). Burchard modelling cyanobacteria in shallow coastal seas. Ecol. Model..

[22] Mazur-Marzec H (2013). Occurrence of cyanobacteria and cyanotoxin in the Southern Baltic Proper. Filam. cyanobacteria versus single-celled picocyanobacteria, Hydrobiol..

[23] Hieronymus J, Eilola K, Olofsson M, Hense I, Meier HEM, Almroth-Rosell E (2021). Modeling cyanobacteria life cycle dynamics and historical nitrogen fixation in the Baltic Proper. Biogeosciences.

[24] Hense I, Beckmann A (2006). Towards a model of cyanobacteria life cycle-effects of growing and resting stages on bloom formation of N_2 fixing species. Ecol. Model..

[25] Munkes B, Löptien U, Dietze H (2021). Cyanobacteria Blooms in the Baltic Sea: A Review of Models and Facts. Biogeosciences.

[26] Neumann T, Fennel W, Kremp C (2002). Experimental simulations with an ecosystem model of the Baltic Sea: A nutrient load reduction experiment. Global Biogeochem. Cycles.

[27] Paerl HW, Huisman J (2009). Climate change: A catalyst for global expansion of harmful cyanobacteria blooms. Environ. Microbiol. Rep..

[28] Viitasalo M, Bonsdorff E (2022). Global climate change and the Baltic Sea ecosystem: direct and indirect effects on species, communities and ecosystem functioning. Earth Syst. Dynam..

[29] Wasmund N (1997). Occurrence of cyanobacteria blooms in the Baltic Sea in relation to environmental conditions. Int. Revue der gesamten Hydrobiologie und Hydrographie.

[30] Lips U (2008). Abiotic factors influencing cyanobacteria bloom development in the Gulf of Finland (Baltic Sea). Hydrobiologia.

[31] Unger J (2013). Response of Nodularia spumigena to pCO$$_2$$ Part 3: Turnover of phosphorus compounds. Biogeosciences.

[32] Rakko A, Seppäälä J (2014). Effect of salinity on the growth rate and nutrient stoichiometry of two Baltic Sea filamentous cyanobacterial species. Estonian J. Ecol..

[33] Wulff A (2018). Ocean acidification and desalination: climate-driven change in a Baltic Sea summer microplanktonic community. Marine Biol..

[34] Falcon LI, Pluvinage S, Carpenter EJ (2005). Growth kinetics of marine unicellular N2-fixing cyanobacteria isolates in continuous culture in relation to phosphorus and temperature. Marine Ecol. Progr. Ser..

[35] La Roche J, Breitbarth E (2005). Importance of the diazotrophs as a source of new nitrogen in the ocean. J. Sea Res..

[36] Mills MM, Arrigo KR (2010). Magnitude of oceanic nitrogen fixation influenced by the nutrient uptake ratio of phytoplankton. Nat. Geosci..

[37] Ploug H, Adam B, Musat N, Kalvelage T, Lavik G, Wolf-Gladrow D, Kuypers MM (2011). Carbon, nitrogen and O$$_2$$ fluxes associated with the cyanobacterium Nodularia spumigena in the Baltic Sea. ISME J..

[38] Landolfi A, Dietze H, Koeve W, Oschlies A (2013). Overlooked runaway feedback in the marine nitrogen cycle: the vicious cycle. Biogeosciences.

[39] Landolfi A, Koeve W, Dietze H, Kähler P, Oschlies A (2015). A new perspective on environmental controls of marine nitrogen fixation. Geophys. Res. Lett..

[40] Löptien, U. & Dietze, H. Contrasting juxtaposition of two paradigms for diazotrophy in an Earth System Model of intermediate complexity. Preprint at https://bg.copernicus.org/preprints/bg-2020-96/ (2020).

[41] Neumann T (2000). Towards a 3D-ecosystem model of the Baltic Sea. J. Marine Syst..

[42] Savchuk OP (2002). Nutrient biogeochemical cycles in the Gulf of Riga: scaling up field studies with a mathematical model. J. Marine Syst..

[43] Eilola K, Meier HEM, Almroth E (2009). On the dynamics of oxygen, phosphorus and cyanobacteria in the Baltic Sea; A model study. J. Marine Syst..

[44] Janssen F, Neumann T, Schmidt M (2004). Inter-annual variability in cyanobacteria blooms in the Baltic Sea controlled by wintertime hydrographic conditions. Marine Ecol. Progr. Ser..

[45] Karlson, B., Eilola, K. & Hansson, M. Cyanobacterial blooms in the Baltic Sea-correlating bloom observations with environmental conditions. *Proc 13th Int Conf on Harmful Algae*, 247-252 (2008).

[46] Kahru M, Elmgren R, Kaiser J, Wasmund N, Savchuk O (2020). Cyanobacterial blooms in the Baltic Sea: Correlations with environmental factors. Harmful Algae.

[47] Dietze H, Löptien U, Getzlaff K (2014). MOMBA 10-a high-resolution Baltic Sea configuration of GFDL’s Modular Ocean Model. Geosci. Model Develop..

[48] Dietze H, Löptien U (2016). Effects of surface current/wind interaction in an eddy-rich general ocean circulation simulation of the Baltic Sea. Ocean Sci..

[49] Lehmann A, Myrberg K (2008). Upwelling in the Baltic Sea-A review. J. Marine Syst..

[50] Vigouroux G (2021). Trend correlations for coastal eutrophication and its main local and whole-sea drivers - Application to the Baltic Sea. Sci. Total Environ..

[51] Stal LJ, Staal M, Villbrandt M (1999). Nutrient control of cyanobacterial blooms in the Baltic Sea. Aquatic Microbial Ecol..

[52] Hansson M, Håkansson B (2007). The Baltic Algae Watch System-a remote sensing application for monitoring cyanobacterial blooms in the Baltic Sea. J. Appl. Remote Sens..

[53] Griffies, S. M. Elements of MOM4p1. GFDL Ocean Group Technical Report No. 6, (NOAA/Geophysical Fluid Dynamics Laboratory, Version 16 December 2009).

[54] Fennel W, Seifert T, Kayser B (1991). Rossby radii and phase speeds in the Baltic Sea. Cont. Shelf Res..

[55] Large WG, McWilliams JC, Doney SC (1994). Oceanic vertical mixing - A review and a model with nonlocal boundary-layer parameterization. Rev. Geophys..

[56] Dietze H, Kriest I (2012). $$^{137}$$Cs off Fukushima Dai-ichi, Japan -model based estimates of dilution and fate. Ocean Sci..

[57] Dietze H, Löptien U, Getzlaff J (2020). MOMSO 1.0-an eddying Southern Ocean model configuration with fairly equilibrated natural carbon. Geosci. Mod. Develop..

[58] Berg P, Döscher R, Koenigk T (2013). Impacts of using spectral nudging on regional climate model RCA4 simulations of the Arctic. Geosci. Mod. Develop..

[59] Böning CW, Cox MD (1988). Particle dispersion and mixing of conservative properties in an eddy-resolving model. J. Phys. Oceanogr..

[60] Glessmer MS, Eden C, Oschlies A (2009). Contribution of oxygen minimum zone waters to the coastal upwelling off Mauritania. Progr. Oceanogr..

[61] Löptien U, Meier HEM (2011). The influence of increasing water turbidity on the sea surface temperature in the Baltic Sea: A model sensitivity study. J. Marine Syst..

[62] Reynolds CS, Oliver RL, Walsby AE (1987). Cyanobacterial dominance: The role of buoyancy regulation in dynamic lake environments. New Zealand J. Marine Freshwater Res..

